# Serine Hydroxymethyltransferase (SHMT) in biology and disease: molecular mechanisms and therapeutic targeting

**DOI:** 10.1186/s12967-026-08044-x

**Published:** 2026-04-21

**Authors:** Jia-Ying Li, Xue-Qing Zhang, Rui Sheng

**Affiliations:** Department of Anesthesiology, The Second Affiliated Hospital of Soochow University, Department of Pharmacology and Laboratory of Aging and Nervous Diseases, Jiangsu Key Laboratory of Drug Discovery and Translational Research for Brain Diseases, College of Pharmaceutical Sciences, Suzhou Medical College of Soochow University, Suzhou, 215123 China

**Keywords:** SHMT, One-carbon metabolism, Cancer, Hepatic metabolism, Inflammation, Neurodegenerative diseases

## Abstract

**Background:**

Serine Hydroxymethyltransferase 1 and 2 (SHMT1/2), as key enzymes in folate metabolism, regulate diverse physiological processes through one-carbon metabolism, including nucleotide biosynthesis, methylation reactions, and redox homeostasis. As a form of metabolic reprogramming, dysregulated SHMT-mediated one-carbon metabolism has been increasingly reported in various human cancers and liver metabolic diseases. Therefore, a concise review of its regulatory roles and disease-associated functions is necessary to clarify its biological and translational significance.

**Main body:**

This review summarizes the molecular functions of SHMT1/2, with particular emphasis on their central roles in the folate-methionine cycle and serine-glycine metabolic pathway. Beyond their canonical enzymatic activities, SHMT1/2 also exhibit critical non-enzymatic functions. Their activity is tightly regulated by transcription factors and post-translational modifications. SHMT1/2 play significant roles in multiple pathological conditions and are implicated in the initiation and progression of various cancers, such as ovarian, lung, liver, gastric, colorectal, and renal carcinomas. Moreover, SHMT-mediated one-carbon metabolism critically influences liver diseases (e.g., non-alcoholic fatty liver disease, steatohepatitis), inflammatory disorders (e.g., skin inflammation, osteoarthritis), and neurological diseases (e.g., schizophrenia, hypomyelinating leukodystrophy, stroke). Finally, this review discusses the clinical translational potential of SHMT inhibitors in cancer-targeted therapies, offering a theoretical basis for expanding the therapeutic applications of SHMT1/2 in diseases associated with dysregulated one-carbon metabolism.

**Conclusions:**

SHMT1/2 regulate diseases associated with dysregulated one-carbon metabolism through both their enzymatic and non-enzymatic functions. Particularly in cancer, they function as either oncogenes or tumor suppressors in a context-dependent manner, holding significant potential as diagnostic, prognostic, and therapeutic targets.

**Graphical Abstract:**

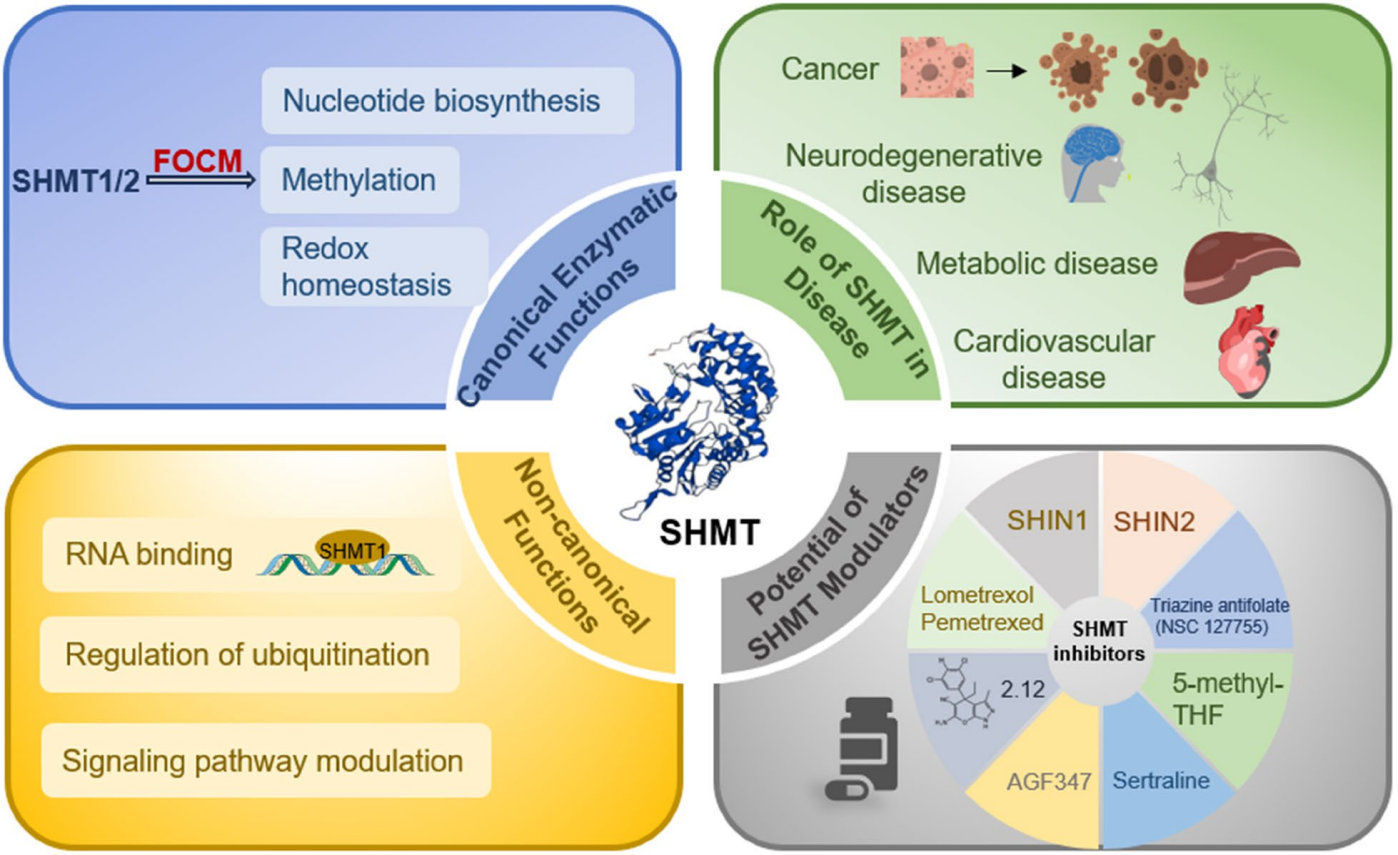

## Introduction

One-carbon (1C) metabolism constitutes a highly conserved metabolic pathway across evolution. This process is critically dependent on the cofactor folate, which mediates the transfer of 1C units essential for fundamental biosynthetic reactions. These reactions encompass nucleotide biosynthesis (purines and thymidylate), diverse methylation processes (including homocysteine remethylation), and redox regulation [1]. 1C metabolism is tightly integrated with serine and glycine metabolism. The primary function of these amino acids is to supply the 1C units necessary for the synthesis of purines, thymidine, and methionine [2, 3]. Given its central role in supporting cellular proliferation and growth, 1C metabolism is implicated in numerous biological phenomena, such as stem cell maintenance and oncogenesis [4]. Furthermore, depletion of serine and glycine disrupts metabolic homeostasis, representing a characteristic feature of metabolic syndromes [5, 6], while dysregulation of 1C metabolism represents one of the most prevalent forms of metabolic reprogramming.

Serine hydroxymethyltransferases (SHMTs), comprising the cytosolic isoform (SHMT1) and mitochondrial isoform (SHMT2), are recognized as pivotal regulators in 1C metabolism [7]. The *de novo* synthesis of serine and glycine initiates with the glycolytic intermediate 3-phosphoglycerate (3-PG). This intermediate undergoes three enzymatic steps catalyzed by phosphoglycerate dehydrogenase (PHGDH), phosphoserine aminotransferase 1 (PSAT1), and phosphoserine phosphatase (PSPH). Collectively, these enzymes generate serine (which can be further converted to glycine via SHMTs) [8, 9]. Serine and tetrahydrofolate (THF) are subsequently converted into glycine and 5,10-methylenetetrahydrofolate (5,10-meTHF) by SHMT1 or SHMT2 [10]. This reversible reaction, catalyzed by SHMT1/SHMT2, enables the supply of 1C units for nucleotide synthesis, and also affects DNA methylation and NADH/NADPH production [2, 11].

Historically, it has been recognized that targeting folate metabolism to disrupt nucleotide synthesis represents an effective therapeutic strategy. Notably, the folate cycle participates in diverse metabolic processes beyond nucleotide production. Recent studies demonstrate that SHMT-mediated 1C metabolism influences cancer progression and remodels the tumor microenvironment (TME) by modulating antioxidant defense, mitochondrial translation, and formate accumulation. These findings underscore the role of folate cycle intermediates in tumor growth, metastasis, and microenvironmental adaptation [12–14]. Concurrently, research demonstrates that defects in 1C metabolism contribute to neurological disorders, including Alzheimer’s disease [15], brain atrophy [16], and ischemic stroke [17, 18]. Based on these findings, this review examines alterations in the flux of SHMT-mediated 1C metabolism in diseases (e.g., cancers, metabolic disorders, and neurological diseases), elucidates the mechanistic roles of SHMT in pathogenesis, and evaluates its therapeutic potential.

## *SHMT* gene and protein structure

SHMT is a metabolic enzyme that uses tetrahydrofolate as a coenzyme. SHMT is present in plant and mammals and plays a crucial part in two major metabolic pathways: 1C metabolism and amino acid metabolism. SHMT catalyzes the reversible conversion of L-serine to glycine, transferring 1C unit to THF to generate 5,10-meTHF. In mammals, two major isoforms are known to exist in SHMT: one is found in the cytoplasm and the other is found in the mitochondria. The *SHMT1* gene is found on the chromosome 17p11.2, while the *SHMT2* gene is found on the 12q13 region [19, 20]. These two genes exhibit a sequence identity of 66%. The splice variant of *SHMT2*, designated as *SHMT2α*, lacks exon 1 due to the absence of the mitochondrial import signals and thus localizes to the cytoplasm [21, 22]. The pseudogene *SHMT-ps1* is located on the short arm of chromosome 1 (1p32.3–p33) and shares 90% sequence identity with the cytoplasmic *SHMT1* cDNA. However, it is inactive because it lacks a promoter sequence and a functional translation start site. Current evidence suggests it is restricted to primates [23].

SHMT functions as a pyridoxal-5′-phosphate (PLP)-dependent enzyme [24] and can catalyze non-THF-dependent reactions, including reversible cleavage of β-hydroxy amino acids, decarboxylation of aminomalonic acid, and racemization/transamination of D- and L-alanine [25, 26]. SHMT mostly exists as a homodimer in prokaryotes, while the eukaryotic SHMT exists as a homotetramer [27]. Eukaryotic SHMT assumes a tetrameric structure, with each subunit existing in dynamic equilibrium between two conformational states: open and closed. The catalytic unit is a dimer, which features PLP-binding sites at the dimer interface. Binding of PLP induces conformational transitions between disordered and open versus ordered and closed states, thereby promoting tetramer assembly. SHMT activity is allosterically regulated by oligomeric state changes. The closed conformation of SHMT1 is sufficiently stable to allow SHMT1 to adopt a tetrameric form in the cytoplasm regardless of PLP availability. In contrast, SHMT2 exists predominantly as a dimer in the open conformation and requires PLP binding to transform into an active tetramer [28].

The human cytosolic SHMT monomer comprises 483 amino acids [29], with each subunit binding one PLP cofactor. The monomeric fold comprises three distinct domains. The N-terminal region (residues 11–53) mediates inter-subunit interactions through two α-helices and a β-strand, stabilizing dimeric or tetrameric assemblies. The large domain (residues 53–321) contains the PLP-binding site as the catalytic center for tetrahydrofolate interconversion. Within this domain, a histidine residue (His137) stabilizes the tetramer via intersubunit stacking interactions. The active site is constituted by residues from both subunits situated at the dimer interface. The C-terminal small domain, which encompasses residues 322–480, undergoes a folding process that results in an *αβ* sandwich [24, 28, 30].

**Fig. 1:**
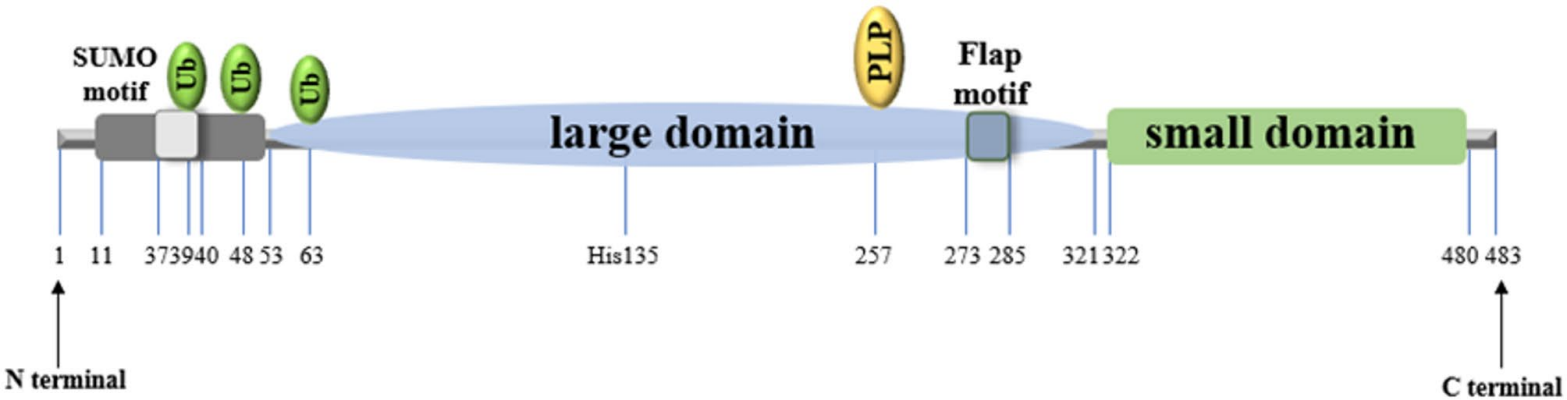
Schematic representation of the structure of human cytoplasmic SHMT (SHMT1). The structure comprises an N-terminal region, a large domain housing the catalytic and RNA-binding sites, and a C-terminal small domain. SUMOylation at the conserved IKKE site, a classic SUMO motif, favors SHMT1's nuclear localization and accumulation over ubiquitination-mediated nuclear export and degradation

SHMT1 contains a 13-amino acid β-hairpin structure (273-VKSVDPKTGKEIL-285), referred to as the “RNA-binding flap,” which is essential for RNA interaction. RNA binding requires both tetramer assembly and the flap motif [31]. SHMT1 also contains a conserved SUMO motif (IKKE), and both SUMO and ubiquitin modifications target the same lysine residue (Lys-39), suggesting a potential mechanism whereby nuclear localization and accumulation of SHMT1 are controlled. Ubiquitination at Lys-39 can mediate SHMT1 degradation. Furthermore, K48-linked ubiquitination in the cytoplasm has been shown to promote SHMT1 degradation, whereas K63-linked ubiquitination in the nucleus prevents degradation by blocking SUMO-2/3 modification, a process mediated by Ubc9 (ubiquitin-conjugating enzyme 9, a SUMO-binding enzyme). Therefore, K63-linked ubiquitination contributes to SHMT1 stabilization. The dynamic interplay between SUMOylation and ubiquitination of SHMT1 has been demonstrated to regulate its subcellular distribution and protein stability [32] (as shown in Fig. 1).

## Functions of SHMT

### SHMT-mediated one-carbon metabolism

#### Folate cycle

It is widely accepted that 1C metabolism is composed of two distinct cycles: the folate cycle and the methionine cycle, with SHMTs serving as key regulators primarily acting within the folate cycle [33]. Folate-mediated One-carbon Metabolism (FOCM) is defined as a metabolic network characterized by extensive compartmentalization, with reactions distributed across the cytoplasm, nucleus, and mitochondria [3]. FOCM regulates cellular functions through multiple mechanisms: it controls the synthesis of nucleotides and specific amino acids, in addition to S-adenosylmethionine (SAM) and glutathione (GSH) [34]; participates in redox balance via GSH production [35]; and not only allocates 1C units to biosynthetic pathways but also integrates nutrient signals by modulating SAM-dependent methylation reactions (epigenetics) and redox status.

**Fig. 2:**
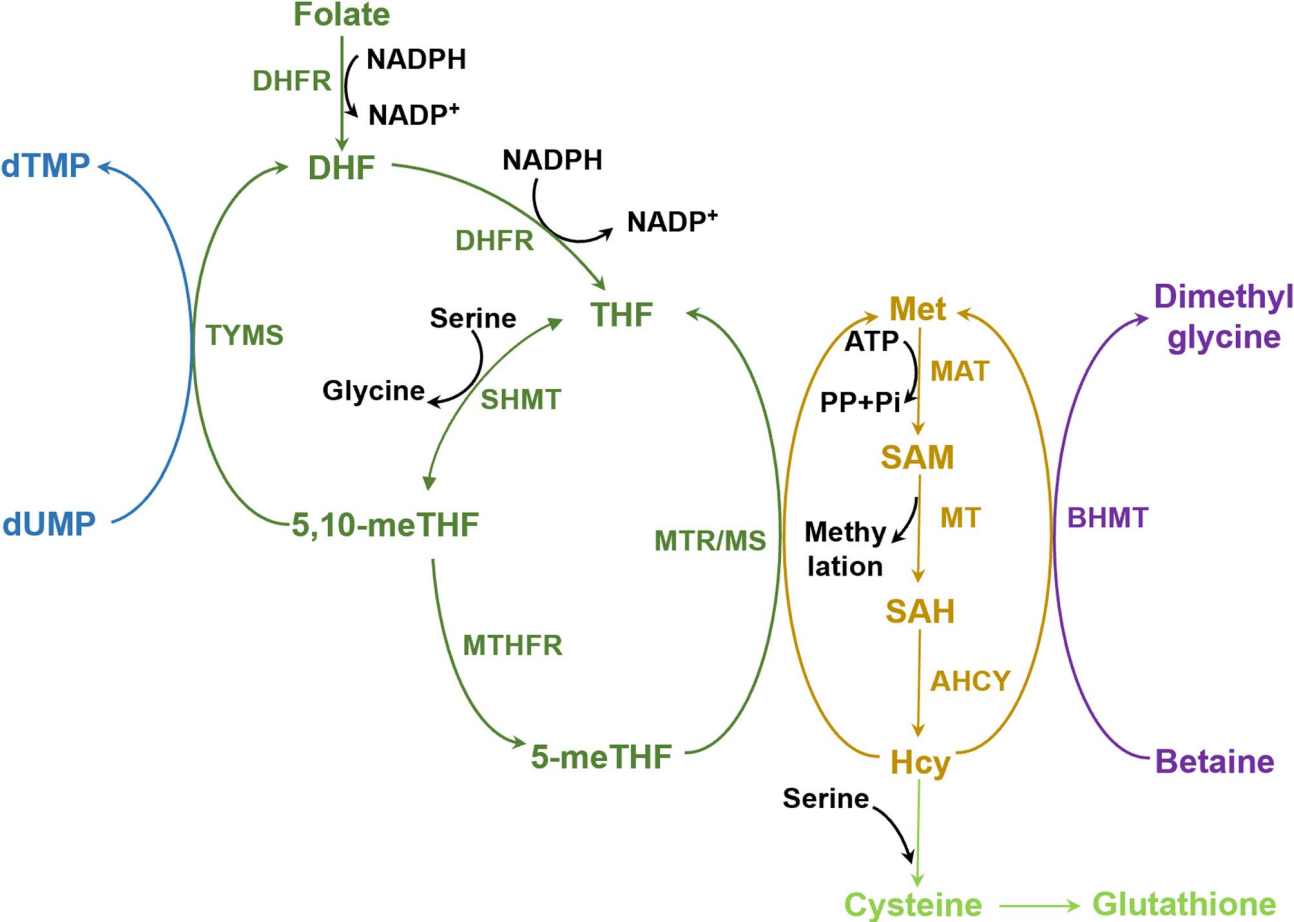
The folate and methionine cycles. Folate is reduced to DHF and THF through an NADPH-dependent reaction mediated by DHFR. THF acts as a 1C unit carrier in the folate cycle, where SHMT converts serine into glycine, transferring a 1C unit to THF to form 5,10-meTHF. 5,10-meTHF is utilized by TYMS to generate DHF while converting dUMP into dTMP, a critical step in DNA synthesis. Concurrently, the folate cycle intersects with the methionine cycle: 5-meTHF and homocysteine are converted into THF and methionine via MS to produce SAM, which is the universal methyl donor for diverse methylation reactions, including lipid methylation. Additionally, homocysteine can be diverted to the transsulfuration pathway to provide cysteine (a precursor of GSH)

Following absorption in the jejunum, folate is sequentially reduced by dihydrofolate reductase (DHFR, which is targeted by methotrexate (MTX)) into dihydrofolate (DHF) and then THF, the biologically-active form of folate within cells. THF, in conjunction with serine, enters the 1C unit pool to participate in various biosynthetic reactions, including the interconversion of serine and glycine, *de novo* purine synthesis, thymidylate (dTMP) synthesis, and remethylation of homocysteine to methionine [36]. SHMT1 or SHMT2 converts serine into glycine, creating 5,10-meTHF. The process of inhibiting mitochondrial SHMT2 results in a disruption of the supply of 1C units, leading to impaired dTMP and dTTP synthesis, cell cycle arrest, and embryonic developmental defects [37]. Notably, the predominant form in physiological folate metabolism is 5-methyltetrahydrofolate (5-meTHF), whereas unmetabolized folic acid (UMFA) constitutes < 10% of total folate [4, 38]. In the methionine cycle, a methyl group is transferred from 5-meTHF to homocysteine through a reaction catalyzed by vitamin B12 (cobalamin)-dependent methionine synthase (MTR/MS), producing methionine and regenerating THF [39, 40] (as shown in Fig. 2). Since mammals cannot synthesize folate, dietary deficiency may lead to fetal neural tube defects and anemia [41].

#### Folate cycle and compartmentalization

Folate metabolism involves two parallel yet complementary pathways within the cell’s cytoplasm and mitochondria. 1C units are essential for cellular growth and proliferation. The novel 1C units under scrutiny in this study are derived from amino acids such as serine, glycine, and choline degradation products dimethylglycine and methylglycine (sarcosine). The glycine cleavage system (GCS) has been identified as a primary source of 1C units, with folate molecules serving as 1C unit carriers [42].

The initial phase in this pathway is catalyzed by enzymes belonging to SHMT family. SHMT1 and SHMT2 independently convert serine to glycine, transferring a 1C unit to THF to generate 5,10-meTHF in their respective compartments. In the cytosol, 5,10-meTHF plays a crucial role in two significant metabolic processes: thymidylate synthesis and the methionine cycle [43]. Within mitochondria, SHMT2 plays a pivotal role in mitochondrial translation. This process relies on the supply of methyl donors, which are essential for the production of taurinomethyluridine [44]. Subsequently, 5,10-meTHF is transformed into the highly oxidized folate species, 10-formyl-THF. In the cytosol, the transformation is mediated by methylenetetrahydrofolate dehydrogenase 1 (MTHFD1), a multifunctional enzyme that exhibits three distinct activities. The following enzymes were identified: 5,10-methylenetetrahydrofolate dehydrogenase, 5,10-methenyltetrahydrofolate cyclohydrolase, and 10-formyltetrahydrofolate synthetase. In mitochondria, the catalytic conversion is carried out by methylenetetrahydrofolate dehydrogenase 2 (MTHFD2) or its homolog MTHFD2-like (MTHFD2L), mitochondrial enzymes harboring both methylenetetrahydrofolate dehydrogenase and methenyltetrahydrofolate cyclohydrolase activities. A comparison of normal cells and cancer cells reveals that the former expresses these enzymes; however, MTHFD2 exhibits notably higher baseline expression levels [45]. The essential nature of cytosolic 10-formyl-THF for *de novo* purine synthesis is indicated by the requirement of two 10-formyl-THF molecule for each newly synthesized purine base [2, 15]. In mitochondria, 10-formyl-THF is required to generate formylmethionyl-tRNA, which initiates mitochondrial translation [46].

**Fig. 3:**
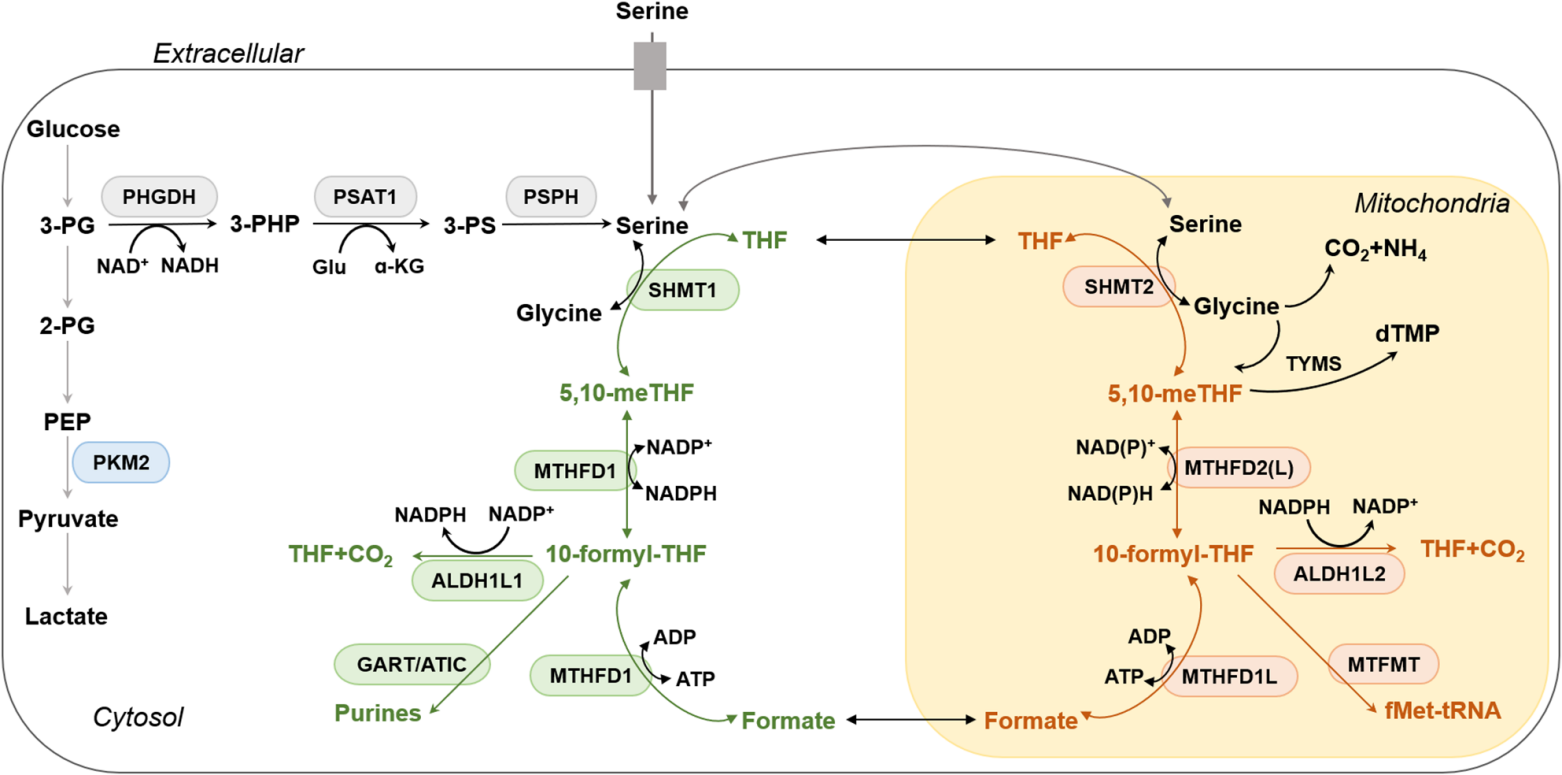
The *De Novo* Serine Synthesis Pathway (SSP) and Compartmentalization of FOCM. The SSP generates serine from the glycolytic intermediate 3-PG through three sequential reactions. First, PHGDH catalyzes the NAD⁺-dependent oxidation of 3-PG to 3-PHP. Second, PSAT1 converts 3-PHP into 3-PS via a glutamate-dependent transamination reaction. Finally, PSPH hydrolyzes 3-PS to produce serine. Serine acts as an allosteric activator of PKM2. Under serine deficiency, reduced PKM2 activity redirects the 3-PG flux toward the SSP. Folate-mediated 1C metabolism occurs in the cytosol and mitochondria through interconnected anabolic reactions. Enzymes such as SHMT, MTHFD1/MTHFD2(L), and ALDH1L1/2 mediate the production of key metabolites, including 5,10-meTHF, 10-formyl-THF, and formate. Mitochondrial 10-formyl-THF initiates mitochondrial translation by producing fMet-tRNA. 5,10-meTHF serves as a substrate for TYMS to produce dTMP. Cytosolic 10-formyl-THF is utilized by GART and ATIC for *de novo* purine nucleotide synthesis

In most cells, 1C metabolism exhibits a directional flux from mitochondria to cytosol [47, 48]. Since 10-formyl-THF cannot traverse mitochondrial membranes, it is hydrolyzed to formate in the cytosol by MTHFD1 or in mitochondria by MTHFD1L (a paralog structurally similar to MTHFD1 but lacking catalytic ability in their respective dehydrogenase and cyclohydrolase domains) [49]. Mitochondrial formate then diffuses into the cytosol to support *de novo* synthesis of purine and pyrimidine (anabolism) or is excreted via formate overflow. Finally, 10-formyltetrahydrofolate dehydrogenase 1 (ALDH1L1) and 2 (ALDH1L2) catalyze the complete oxidation of 10-formyl-THF to CO_2_ in the cytosol and mitochondria, respectively, while generating NADPH (as illustrated in Fig. 3).

#### SHMT and serine-glycine metabolism

Serine and glycine are non-essential amino acids (NEAAs) that can be interconverted. Both support diverse metabolic pathways, including phospholipid biosynthesis (e.g., sphingolipids, phosphatidylserine), GSH synthesis, porphyrin production, creatine metabolism, and purine/pyrimidine synthesis. Serine also functions as the primary 1C unit donor in the folate cycle. Serine is a compound that can be obtained from the environment via specialized transporters or synthesized *de novo* via the serine synthesis pathway (SSP). Firstly, the glycolytic intermediate 3-PG is oxidized to 3-phosphohydroxypyruvate (3-PHP) through the action of PHGDH, coupled with NAD⁺ reduction to NADH. Then, the conversion of 3-PHP to 3-phosphoserine (3-PS) is facilitated by glutamate-dependent transamination, a process that is accelerated by PSAT1. Finally, PSPH hydrolyzes 3-PS to generate serine (as shown in Fig. 2). Serine has been demonstrated to function as an allosteric activating agent for the pyruvate kinase M2 isoform (PKM2). Under serine deficiency, a decrease in PKM2 activity causes the flux of 3-PG to be redirected toward SSP [50]. 2-phosphoglycolate (2-PG) inhibits PHGDH, exerting negative feedback regulation on the SSP [51]. In mitochondria and cytosol, serine is catabolized to glycine via SHMT1/2, while cytosolic SHMT1 also synthesizes serine by consuming glycine and 1C units (observed in HEK293T and HCT-116 cells) [52]. Glycine functions as a precursor in the biosynthesis of GSH, purines, creatine, and heme. Glycine is acquired by cells directly from serum or is derived from choline/serine catabolism. When extracellular glycine is limited, serine-derived glycine synthesis becomes essential for the production of purines and GSH [53].

The principal function of serine and glycine is to supply 1C units for the synthesis of purines, thymidine, and methionine [54]. This occurs via two pathways: (1) the process of converting serine to glycine, along with the release of a 1C unit, is catalyzed by SHMT, or (2) The GCS breaks down glycine into CO₂ and a 1C unit [4]. Collectively, these pathways are termed serine-glycine-one-carbon (SGOC) metabolism. Serine and glycine have been demonstrated to play a pivotal role in maintaining redox balance by synthesizing GSH and NADPH, which is dependent on folate. GSH requires three amino acids—glutamate, cysteine, and glycine—wherein glycine is produced via the folate cycle and cysteine via the methionine cycle. Under oxidative stress, a shift in serine-dependent 1C metabolism occurs from methylation to transsulfuration, leading to the condensation of serine with homocysteine to generate cysteine, thus enhancing GSH synthesis [55, 56] (see Fig. 1). The catabolism of serine in mitochondria and its synthesis in the cytosol form a thermodynamically driven cycle. The cycle’s regulation is enabled by the electrochemical gradient between mitochondrial NAD^+^/NADH and cytosolic NADPH/NADP^+^ [57]. An elevated cytosolic NADPH/NADP⁺ ratio has been demonstrated to promote the flux of formate toward cytosolic serine synthesis, while the homeostasis of serine-glycine interconversion critically depends on SHMT-mediated regulation.

### Non-enzymatic functions of SHMT

Historically, recognition of SHMT has centered on its enzymatic role in catalyzing the interconversion of serine and glycine and mediating 1C unit transfer. However, recent studies have unveiled additional non-enzymatic functions of SHMT, including RNA binding, regulation of protein ubiquitination, and direct participation in signaling pathways that play critical roles in metabolic reprogramming and disease pathogenesis.

Research indicates that SHMT can bind to RNA. SHMT1 inhibits the translation of SHMT2 by directly binding to the 5’UTR (UTR2) of the SHMT2 transcript. It has been found that the formation of the SHMT1-UTR2 complex can inhibit the serine hydroxymethyltransferase activity of SHMT1 in the direction of glycine generating serine, leading to the accumulation of glycine in cells. SHMT1 exerts its post-transcriptional regulatory effect directly through “protein-RNA” interactions and is independent of its enzymatic activity [58]. Furthermore, SHMT2 also regulates amyloid precursor protein (APP) metabolism mediated by ADAM10 (ADAM metallopeptidase domain 10) through RNA binding. Specifically, SHMT2 can directly bind to the GAGGG motif in the 5’UTR of *ADAM10* mRNA to enhance its translation efficiency and enzyme activity, promoting the non-amyloid pathway processing of APP, ultimately reducing β-amyloid (Aβ) deposition [59] and enhancing cognitive abilities in mouse models of Alzheimer’s disease [60].

In addition to RNA binding, it has been demonstrated that SHMT exerts a regulatory function in the context of protein interactions. SHMT2, a component of the BRCC36 isopeptidase complex (BRISC), has been identified as a critical protein involved in deubiquitination of the BRISC-targeted substrate, interferon alpha receptor 1 (IFNAR1). BRISC-SHMT2 has been shown to disassemble the K63-ubiquitin chain on IFNAR1, thereby limiting receptor endocytosis and lysosomal degradation and regulating protein turnover of IFNAR1, independently of the catalytic activity of SHMT2. Notably, the SHMT2 dimer has been shown to act as a reversible endogenous BRISC inhibitor, blocking non-specific deubiquitinating enzyme (DUB) activity. This function was initially defined as a non-enzymatic role of SHMT2 by Miriam Walden’s team [61, 62]. Notably, SHMT1 and SHMT2α (SHMT2α is a splice variant of SHMT2) function as scaffolding proteins during DNA replication to exert their regulatory effects, and are localized in the cell nucleus during the S phase along with TYMS and DHFR as part of the thymidine cycle. This process is essential for the synthesis of nuclear dTMPs, thereby preventing uracil misincorporation into DNA [63, 64]. In the context of gastric cancer, SHMT2 reduces the ubiquitination degradation of hypoxia-inducible factor-1α (HIF-1α) through direct protein-protein interactions with non-enzymatic functions, enhances its protein stability, and subsequently activates the VEGF-STAT3 pathway to promote tumor proliferation. However, the precise mechanism by which this occurs is not yet fully established [65]. In the context of colorectal cancer (CRC), studies have observed that SHMT2 directly interacts with β-catenin in the cytoplasm, utilizing a lysine residue at position 64 to impede the process of ubiquitination-mediated degradation of β-catenin. This interaction serves to regulate the expression of downstream target genes. Concurrently, β-catenin has been shown to promote the transcription of SHMT2, facilitated by the action of the transcription factor TCF4. This process establishes a positive feedback loop that contributes to the promotion of cancerous growth [66].

## Regulation of SHMT

### Transcriptional regulation of SHMT

In consideration of the pivotal functionality of 1C metabolism, elucidating the regulatory mechanisms of SHMT is a major focus in metabolic research. Vitamin B6-coupled carriers suppress SHMT1 function to disrupt thymidylate biosynthesis in cancer cells, thereby inhibiting tumor growth [67]. Myc, as an oncogenic transcription factor, can directly bind to the E-box sequence in the promoter region of the SHMT2 gene, significantly upregulating the mRNA and protein levels of SHMT2 [68]. RNA-seq data from the TCGA database show that SHMT2 is highly expressed in many cancer types and that its expression level is significantly and positively correlated with Myc amplification/overexpression. Further experiments have confirmed that Myc promotes the 1C metabolism pathway through SHMT2 by providing 1C units for purine and thymidylate synthesis, which supports the rapid proliferation of cancer cells [21]. Both in vitro cell experiments and in vivo animal models have confirmed that Myc promotes cancer cell proliferation, migration, and tumor growth by up-regulating SHMT2, and knocking down SHMT2 can reverse this effect [68, 69]. SHMT2 is subject to regulation by Myc and, in turn, exerts a regulatory influence on Myc through epigenetic mechanisms, thereby establishing a positive feedback loop that serves to amplify the metabolic reprogramming and proliferation capacity of tumors [70]. Nuclear enrichment of glycogen synthase kinase 3 (GSK3) has been demonstrated to exert a substantial repression effect on the transcription and expression of genes implicated in *de novo* serine synthesis, including SHMT2 and MTHFD2 [1]. Nuclear factor erythroid 2-related factor 2 (NRF2) and its repressor, KEAP1, have been demonstrated to exert regulatory functions in serine/glycine metabolism. This regulation occurs through activating transcription factor 4 (ATF4), which in turn controls the expression of key serine/glycine biosynthetic enzymes, including PHGDH, PSAT1, and SHMT2. These enzymes are involved in the synthesis of essential molecules that support the production of GSH and nucleotides [71]. Insulin-like growth factor 2 mRNA-binding protein 1 (IGF2BP1) enhances SHMT2 expression by stabilizing its mRNA [72]. Intriguingly, the present study investigates the mechanism by which SHMT1 controls SHMT2 expression, finding that it does so by binding to the 5’-untranslated region (5’-UTR) of the SHMT2 transcript. It is evident that the formation of the SHMT1-UTR2 complex suppresses the serine cleavage activity of SHMT1 [58]. Additionally, elevated non-canonical miRNA-6778-5p plays an instrumental role in the malignant properties of gastric cancer (GC) with low Drosha expression. Evidence indicates that miR-6778-5p exerts feedback regulation over the expression of its host gene, SHMT1, by targeting YWHAE. This process facilitates the mediation of compensatory activation of the cytosolic carbon metabolism, which is critical for sustaining the proliferation of gastric cancer stem cells (GCSCs) [73]. Structurally, the riboregulation of SHMT1 involves RNA acting as an allosteric switch by competing with folate [31]. Modeling of SHMT1 riboregulation reveals dynamic shifts in serine and glycine levels across various cellular compartments [74].

### Post-translational modifications (PTMs) of SHMT

The biological functions of SHMT are not only dictated by its mRNA and protein expression levels but are also dynamically regulated by a plethora of PTMs, such as ubiquitination, phosphorylation, acetylation, and succinylation, which collectively determine its critical roles in cellular metabolism. These modifications form a complex regulatory network by influencing the stability, subcellular localization, enzymatic activity, and non-canonical functions of SHMT isoforms.

Specifically, mutation of the conserved SUMO motif in SHMT1 prevents its translocation to the nucleus [75]. Ubc13-mediated ubiquitination is required for the nuclear export of SHMT1 and enhances its stability within the nucleus, whereas Ubc9-mediated SUMO2/3 modification is involved in its nuclear degradation. SUMO and ubiquitin modifications determine the nuclear localization and accumulation of SHMT1 [32]. Additionally, the microtubule-associated C4HC3-type E3 ligase, MEL, was found to promote SHMT1 degradation through the 26 S proteasome pathway, resulting in a reduction of endogenous SHMT1. Degradation of SHMT1 affects its enzymatic function, thereby initiating plant immune signaling cascades, including enhanced mtROS production [76]. SIRT3 mediates the deacetylation of SHMT2 at the K95 site, thereby disrupting its functional tetrameric structure and inhibiting its enzymatic activity. This process occurs through the K63-ubiquitin-lysosome pathway in a glucose-dependent manner, thereby promoting SHMT2 degradation, reducing serine consumption, lowering NADPH levels, and inhibiting CRC cell proliferation and tumor growth [77]. Additionally, SIRT5 induces desuccinylation of SHMT2 at lysine 280, thereby activating its enzymatic activity and accelerating serine catabolism and proliferation in cancer cells [78]. The above-mentioned three modifications—ubiquitination, deacetylation, and succinylation—exert inhibitory effects on enzymatic function.

SHMT2 is a fatty acylated protein that undergoes defatty-acylation catalyzed by histone deacetylase 11 (HDAC11); although this modification does not alter the enzymatic activity of SHMT2, it affects its ability to regulate the ubiquitination and cell-surface levels of the type I interferon receptor, thereby influencing the non-enzymatic function of SHMT2 in modulating protein ubiquitination [79]. In cancer cells, the upregulation of SHMT2 primarily results from enhanced protein stability, a process regulated by phosphorylation. The MAPK1/PTPMT1 axis synergistically modulates the phosphorylation and stability of SHMT2: MAPK1 phosphorylates SHMT2 at Ser90, enhancing its stability and promoting tumorigenesis, whereas PTPMT1 dephosphorylates the same residue, rendering it susceptible to STUB1-mediated ubiquitination and degradation. This phosphorylation does not affect enzymatic activity, and inhibiting this phosphorylation does not impact global DNA or protein methylation but rather reduces the m^6^A modification of global RNA, thereby suppressing tumor progression [80]. The above-mentioned two modifications—defatty-acylation and phosphorylation—do not affect enzymatic activity but instead influence its non-enzymatic functions.

## Role of SHMT in disease

### SHMT and cancer

SHMT-mediated 1C metabolism influences cancer progression through multiple mechanisms, including modulation of DNA/protein synthesis, redox homeostasis, and methylation reactions. DNA comprises four nucleotide bases: two pyrimidines and two purines. In 1C metabolism, the metabolites dTMP and 10-formyl-THF are critical for *de novo* synthesis of pyrimidines and purines [81]. The rapid proliferation of cancer cells requires abundant DNA nucleotides, making folate metabolism-derived metabolites critical for DNA synthesis. To initiate mitochondrial translation, 10-formyl-THF must be present, as it is required for generating formylmethionyl-tRNA—indispensable for protein synthesis [46]. During metastasis, cancer cells produce high levels of reactive oxygen species (ROS). NADPH, generated through folate metabolism, is vital for maintaining redox homeostasis [14, 82]. SHMT-driven 1C metabolism produces substantial glycine, which functions as a precursor for the synthesis of GSH, further participating in reductive and oxidative reactions [10, 83]. Under hypoxia, the mitochondrial enzyme SHMT2 induces serine catabolism, a process that is imperative for sustaining NADPH production and redox balance. This, in turn, facilitates the survival and growth of tumor cells [68]. Consequently, SHMT-mediated 1C metabolism has evolved into a viable therapeutic target in the field of oncology.

Both SHMT1 and SHMT2 are implicated in cancer pathogenesis, with their expression typically upregulated during tumorigenesis. In primary epithelial ovarian cancer (EOC) specimens, SHMT1 and SHMT2 expression is markedly elevated, and combining chemotherapy with SHMT inhibitors demonstrates potent antitumor efficacy [84]. Non-small cell lung cancer (NSCLC) that exhibits concurrent KRAS and tumor suppressor LKB1 mutations (KL NSCLC) is notoriously treatment-resistant and associated with poor prognosis [85]. Loss of LKB1 enhances SGOC metabolism via SHMT1 and SHMT2; conversely, inhibiting either SHMT1 or SHMT2 induces ROS accumulation and impairs the survival of KLK NSCLC (KEAP1: KLK oncogenotype) [12].

#### The role of SHMT2 in cancer

Among the two SHMT isoforms, SHMT2 exerts greater functional significance. Genetic deletion of *SHMT2* or the GCS disrupts folate/1 C metabolism, leading to developmental abnormalities [86, 87]. Additionally, SHMT2 is recognized as a critical regulator of T-cell activation and cancer cell proliferation [85, 88]. SHMT2 expression is documented in various forms of cancer, such as breast, liver, gastric, colorectal, oral squamous cell carcinoma, bladder and renal cancers [65, 89–94]. In some cancers, SHMT2 overexpression promotes cancer cell proliferation, invasion, and tumorigenesis, while SHMT2 knockdown inhibits tumor progression [77, 95, 96]. In the context of the ischemic tumor microenvironment characteristic of gliomas, the expression of SHMT2 promotes the survival of cancer cells. SHMT2 is elevated in a subset of cancer cells and drives metabolic alterations, thereby enabling cells to survive in the ischemic tumor microenvironment [13].

SHMT2 exerts pro-tumorigenic effects through multiple mechanisms. SHMT2 regulates mitochondrial tRNA methylation by supplying 1C units, thereby mediating the translation of respiratory chain proteins [44, 97]. SHMT2 also promotes lymphomagenesis by modulating DNA and histone methylation to silence tumor suppressors [98]. The inhibition of SHMT2 has been shown to disrupt the TCF3 transcriptional survival program by significantly reducing intracellular glycine and formate levels. This, consequently, inhibits the mTOR pathway and triggers the autophagic degradation of the oncogenic transcription factor TCF3 [99]. SHMT2 further contributes to redox balance. In a HeLa cell xenograft model, SHMT2 expression is associated with the expression of mitochondrial respiratory complex proteins [100]. By maintaining redox homeostasis, SHMT2 supports bladder cancer cell proliferation and suppresses ROS-dependent mitochondrial apoptosis [55]. Notably, hypoxia induces SHMT2 expression, and SHMT2 is essential for sustaining redox equilibrium and cell survival under hypoxic conditions [101, 102]. Furthermore, SHMT2-mediated mitochondrial serine metabolism also drives drug resistance in CRC cells [103]. In CRC, SHMT2 has been shown to bind competitively to the cytosolic p53 protein, thereby sequestering the E3 ubiquitin ligase HDM2(Human double minute 2), thereby inhibiting autophagy and conferring resistance to 5-fluorouracil (5-FU) chemotherapy [104]. Beyond metabolic roles, SHMT2 has been shown to stabilize β-catenin by suppressing its ubiquitination-mediated degradation, fueling CRC progression and metastasis [66].

The mechanisms and pathways through which SHMT2 promotes cancer have been widely studied in subcutaneous xenograft models, and further, the inhibitory effect of SHMT2 on tumor growth was observed in PDX models. The proteomic comparison between tumors and normal patient tissues revealed that SHMT2, which is involved in single-carbon metabolism, was elevated, making it a potential therapeutic target. Common antidepressants and SHMT2 inhibitor sertraline showed anti-tumor efficacy against SETTLE (Spindle Epithelial Tumor with Thymus-Like Differentiation) PDX in preclinical tests [105]. In PDX models of CRC, the combined use of CQ and 5-FU significantly suppressed tumor growth in mice bearing SHMT2-low tumors [104]. In the breast cancer PDX model, the combined use of SHIN1 with chemotherapy drugs (such as paclitaxel) showed a more significant effect compared to single drug treatment. This confirmed that SHMT2 is a potential therapeutic target for breast cancer metastasis. SHMT2 enhances the migration and invasion abilities of breast cancer cells by binding to and upregulating HAX1 (a pro-invasive protein) [106].

#### The role of SHMT1 in cancer

Concurrently, studies indicate that SHMT1 is also overexpressed in certain cancers, and its knockout suppresses cancer cell proliferation [107]. In lung cancer cells, SHMT1 knockdown induces cell cycle arrest, leading to p53-dependent apoptosis. This apoptotic effect is independent of serine or glycine deprivation and is instead attributable to heightened uracil synthesis during DNA replication [108]. SHMT1 contributes to low-grade glioma (LGG) progression by activating the mTORC1 signaling pathway, promoting LGG cell proliferation, invasion, and migration [109]. Evidence shows that SHMT1 heterozygosity results in impaired folate-dependent thymidylate synthesis capacity and modifies Apc^min^ -driven intestinal cancer risk. Although SHMT1 is a serine catabolic enzyme, this study observed no significant impact on methylation capacity [64]. In the presence of cytosolic 1C stress, SHMT1 has the capacity to reverse flux direction, thereby generating 1C units. In instances of hypoxia or electron transport chain inhibition, this adaptation plays a critical role. Elevated NADH/NAD^+^ ratios impede mitochondrial 1C flux, resulting in impaired metabolic processes within the cell. In the context of mitochondrial pathway deficiency, serine catabolism through the SHMT1 pathway becomes the predominant method of 1C acquisition in tumors, which is crucial for xenograft formation [52]. Mitochondrial folate metabolism is not the sole source of 1C units for tumor cells. Cancer cells exhibit significant diversity in utilizing cytoplasmic and mitochondrial folate cycles, with the cytoplasmic pathway being the primary source of 1C units in some tested cancer cell lines. In cancer cells with sufficient folate supply, SHMT2 in the mitochondria plays a dominant role in 1C metabolism, thereby ensuring a continuous supply of formate and NAD(P)H to support biosynthesis and antioxidant processes. In contrast, in cells with reduced folate levels or SLC19A1 deficiency, SHMT1 activity becomes the main driver for 1C metabolism, as mitochondrial flux is impaired [53]. Isocitrate dehydrogenase 3α (IDH3α) modulates 1C metabolism by colocalizing and interacting with SHMT1, thereby promoting glioblastoma progression in an orthotopic glioma mouse model [110]. Separately, SHMT1 promotes CRC liver metastasis (CRLM) by inhibiting AMPK signaling through a formate-mediated mechanism [111]. Furthermore, in bladder cancer, SHMT1 enhances the proliferation, invasion, and epithelial-mesenchymal transition (EMT) of cancer cells while suppressing apoptosis by promoting ATIC and inhibiting the AKT/FOXO3A signaling pathway, collectively driving malignant progression [112].

However, in contrast to its well-established oncogenic functions, SHMT1 acts as a tumor suppressor in some cancers. SHMT1 induces G2/M phase arrest, accompanied by decreased cyclin levels and increased DNA damage markers. The transcription factor HOXD8 inhibits renal cell carcinoma (RCC) cell proliferation and migration by enhancing SHMT1 expression [113]. Similarly, the expression of SHMT1 is markedly diminished in patients diagnosed with hepatocellular carcinoma (HCC), and this reduction in expression is correlated with adverse clinicopathological characteristics and a poor prognosis. SHMT1 suppresses tumor metastasis by inhibiting NADPH oxidase 1 (NOX1)-mediated ROS production [14].

A review of the extant literature reveals a compelling body of evidence that suggests SHMT1 and SHMT2 play instrumental roles in various neoplasias by modulating 1C metabolism. SHMT2 primarily drives tumor progression by maintaining redox balance, supporting mitochondrial function, and mediating epigenetic regulation, whereas SHMT1 is crucial for nucleotide synthesis and metabolic adaptation, with its function exhibiting significant cancer-type specificity. These findings collectively establish SHMT isoforms as highly promising metabolic targets in cancer therapy.

#### Compensatory pathways in SHMT-targeted cancer therapy

Although inhibition of SHMT affects the expression of various metabolites in 1C metabolism, thereby influencing tumor occurrence and progression, metabolic redundancy remains a core mechanism of therapeutic resistance in cancer. When SHMT is inhibited, tumor cells can maintain the supply of 1C units by activating alternative metabolic pathways. The first among these is the mutual compensation between SHMT1 and SHMT2 [63]. Loss of SHMT2 activity is accompanied by compensatory reversal catalyzed by SHMT1, along with the synthesis of 1C units and glycine from serine. The mitochondrial 1C pathway is consistently hyperactivated in cancer, and its loss renders cells dependent on extracellular serine for generating 1C units and on extracellular glycine for GSH synthesis. HCT-116 colon cancer xenografts lacking mitochondrial 1C pathway activity generate the 1C units required for cytosolic serine catabolism [52]. After SHMT2 knockdown, an increase in SHMT1 expression was detected in lung cancer cells, and SHMT2 depletion increases SHMT1-mediated dTTP synthesis [114]. These findings suggest that the compensation of serine and 1C metabolism is achieved, at least in part, through the increased catalysis mediated by cytosolic SHMT1 [53]. When SHMT1 was knocked down in lung cancer cells, SHMT2 and SHMT2α were selectively upregulated [108]. Consistent with these findings, subsequent research also revealed that overexpression of SHMT1 in lung cancer cells leads to a significant decrease in SHMT2 expression, confirming the existence of cross-regulation between the two isoforms, with SHMT1 appearing to suppress SHMT2 expression [115].

Secondly, the purine salvage synthesis pathway compensates for SHMT inhibition. Blockade of succinate dehydrogenase (SDH) elevates succinate and induces SHMT2 succinylation, leading to reduced formate production; in response, cancer cells activate the purine salvage synthesis pathway, a metabolic compensatory adaptation that represents a therapeutic vulnerability [116]. This constitutes a key resistance mechanism while simultaneously creating a targetable metabolic weakness. However, there are currently no specific studies on the compensatory effects of other metabolic pathways in response to SHMT inhibition, and further exploration is needed.

### SHMT and metabolic diseases

As the primary site for 1C metabolism, the liver fulfills a crucial function in the conversion of glycine to serine. Serine is then transformed into pyruvate by serine dehydratase and oxidized in the tricarboxylic acid cycle. Serine and glycine serve as primary carriers in 1C unit transfer, and a large body of research concentrating on circulating metabolites has shown that low glycine is a predictive biomarker for metabolic dysfunction-associated steatohepatitis (MASH) and fibrosis in patients [117]. In fact, appropriate glycine homeostasis is closely linked to metabolic health. Existing research indicates that patients with obesity, Type 2 diabetes, and non-alcoholic fatty liver disease exhibit lower circulating glycine concentrations, with a reported 10–20% reduction [118]. More broadly, glycine concentration is inversely correlated with BMI (Body Mass Index), and weight-loss interventions consistently elevate glycine levels, tending to restore them to healthy ranges [119, 120]. Despite the existence of systematic studies in rodents and patients, the question of whether impaired *de novo* synthesis or elevated catabolism is responsible for glycine depletion remains unresolved [121, 122]. Studies have revealed that the net effect of systemic SHMT flux in rodent models is glycine consumption rather than production, and blocking the key folate enzyme SHMT2 leads to systemic glycine accumulation. This indicates that glycine homeostasis requires reverse SHMT flux [123]. Additionally, hepatic glycine concentrations negatively correlate with vitamin B-6 intake, and insufficient intake may disrupt SHMT1 and SHMT2-mediated 1C metabolism [124]. Genome-scale metabolic models of hepatocytes have revealed serine deficiency in patients with non-alcoholic fatty liver disease (NAFLD), while the expression levels of SHMT1 and SHMT2 are significantly down-regulated in the livers of non-alcoholic steatohepatitis (NASH) patients, and PSPH, SHMT1, and BCAT1 (branched-chain amino acid transaminase 1) have been identified as potential therapeutic targets for NASH. Increasing serine levels in hepatocytes either through dietary serine supplementation or by activating SHMT1/2 to convert glycine to serine may be beneficial for NASH patients [125]. In mouse models of metabolic dysfunction-associated steatotic liver disease (MASLD), glycine levels are low, and the promotion of serine synthesis from glycine via reverse SHMT flux is the underlying cause of glycine depletion in fatty liver. Increasing glycine levels through dietary supplementation or liver-specific SHMT knockdown can promote *de novo* GSH synthesis, thereby alleviating acetaminophen-induced hepatotoxicity [10].

Besides glycine metabolism, 1C metabolism is also intimately associated with hepatic lipid and phospholipid homeostasis, and its dysregulation is a contributing factor in the pathophysiology of MASLD [126]. Specifically, serine catabolism produces hepatic NADPH and maintains hepatic lipogenesis, with the generation of NADPH occurring via the SHMT1-MTHFD1-ALDH1L1 reaction cascade. Furthermore, SHMT1/2 inhibition reduces hepatic lipogenesis, thus highlighting the unique connectivity of hepatic folate metabolism in supporting cytosolic NADPH production and lipogenesis [127]. In light of these findings, SHMT has garnered significant attention as a promising therapeutic target. Furthermore, the crystal structure of human serine hydroxymethyltransferase (hSHMT1/2) interacting with endogenous glycine-conjugated secondary bile acids, combined with mutational analysis, biophysical measurements, and structure-activity relationship studies, demonstrates specific interactions between hSHMT and its ligands, suggesting that secondary bile acid conjugates might function as modulators of SHMT activity [128].

In summary, SHMT orchestrates serine-glycine metabolism and lipogenesis, playing a central regulatory role in liver metabolic homeostasis and serving as an important therapeutic target for metabolic diseases.

### SHMT and neurological disorders

Impairments in 1C metabolism are linked to neurodevelopmental disorders and neurological diseases, manifesting as neuronal apoptosis, DNA damage, mitochondrial dysfunction, and oxidative stress [129]. 1C metabolism-dependent vitamin folate (B9) is imperative for optimal development. Its deficiency during pregnancy and early childhood has been linked to various adverse developmental outcomes, which include neural tube defects [130]. Deficiency of *Shmt1/2* leads to severe phenotypes in the optic lobe, morphological defects in the neuroepithelium, and impaired formation of the basal groove, indicating that *Shmt1/2* is indispensable for normal neuroepithelial development and revealing a mechanistic role in brain development [131]. Studies indicate that polymorphisms in folate-related genes, including *SHMT1/2*, exhibit complex associations with neural tube defects [132]. Inhibition of SHMT2 catalytic activity during embryonic development leads to insufficient dTTP supply and replication stress during the first mitosis, resulting in failure of pronuclear fusion and developmental arrest [37]. In *SHMT2* knockout mice, reduced mitochondrial translation leads to mitochondrial respiratory defects and embryonic lethality, which is attributed to the epigenetic regulation of nuclear genes, including *SHMT2* [86]. Similarly, downregulation of SHMT2 disrupts the balance of glycine/serine metabolism, compromising mitochondrial 1C unit supply in proliferating cells. This imbalance underlies developmental abnormalities such as microcephaly and polymicrogyria [133, 134].

Multiple sclerosis (MS) is a prominent demyelinating disease of the central nervous system, and genone-wide association studies have identified *SHMT1* as a newly discovered susceptibility gene. Aberrant *SHMT1* expression may disrupt methyl donor homeostasis, contributing to MS progression. Therefore, the identification of *SHMT1* as a risk factor for MS further highlights epigenetic regulation via methylation as a key focus in MS susceptibility [135]. Hypomyelinating leukodystrophies (HLDs) are a collective of genetically heterogeneous neurodegenerative disorders distinguished by dysmyelination in the central nervous system, that arises from loss-of-function mutations in pyrroline-5-carboxylate reductase 2 (PYCR2). Notably, PYCR2 deficiency upregulates SHMT2, which mediates glycine synthesis; while knockdown of SHMT2 normalizes glycine levels, thereby ameliorating axonal abnormalities in PYCR2-deficient neurons, positioning SHMT2 as a potential therapeutic target [129]. Clinical genetic analysis of patients revealed that impairment of the mitochondrial 1C metabolic enzyme *SHMT2* leads to a distinct neurodevelopmental disorder, syndromic encephalopathy, and dyskinesia accompanied by cardiac defects [136].

Interestingly, elevated plasma serine levels and abnormal *SHMT1/2* activity were first reported in patients with severe or atypical psychoses in 1984 [137]. The increased expression of *SHMT1* in the brains of schizophrenia patients may represent a genetic component regulating prepulse inhibition (PPI) in mice, implicating its role in the PPI deficits associated with the disease pathogenesis [138].

The findings, when considered collectively, emphasize the critical and multidimensional functions of SHMT isoforms in both neurodevelopment and the maintenance of neurological function, highlighting their broader significance beyond cellular metabolism.

### SHMT and cardiovascular diseases

#### SHMT and cardiac disorders

Congenital heart disease (CHD), a prevalent birth defect, correlates with dysregulated FOCM. Analysis of a cohort of 823 CHD patients revealed that SHMT1 gene polymorphisms significantly interacted with plasma folate levels to influence cardiac development [139]. Genetic polymorphisms in cytoplasmic SHMT (cSHMT, SHMT1), a pivotal enzyme regulating folate homeostasis, have been demonstrated to augment the contribution of the *MTHFR* 677 C→T CT and TT genotypes to cardiovascular disease (CVD) risk [140]. Cardiac autonomic dysfunction is also linked to *cSHMT* metabolic polymorphisms; the C1420T variant reduces methionine cycle activity, altering heart rate variability (HRV) [141]. Mitochondrial SHMT2 deficiency is identified as a causative agent for a novel brain-heart syndrome, characterized by peripheral neuropathy and hypertrophic cardiomyopathy or atrial septal defects in 80% of patients [136].

Beyond embryonic development, *SHMT* dysregulation contributes to acquired cardiovascular diseases. Medial vascular calcification is significantly associated with cardiovascular morbidity and mortality in individuals suffering from chronic kidney disease (CKD). In addition, evidence indicates that SHMT1 knockdown promotes bone/chondrocyte-forming transdifferentiation of vascular smooth muscle cells (VSMCs) into bone and cartilage-forming cells. This process is accompanied by an escalation in oxidative stress within the cells, thereby inducing osteoinductive signaling that is contingent upon oxidative stress. This exacerbates vascular calcification in vitro under hyperphosphatemic conditions [142]. Furthermore, SHMT2 directly influences the energy production of cardiomyocytes by means of modulation of the 1C unit metabolic network. Dickkopf 1 (DKK1) regulates SHMT2 transcription through the AKT-Sp1 (Specificity Protein 1) signaling axis, modulates redox homeostasis and mitochondrial function, and exerts anti-apoptotic and proliferative effects on human PAECs (hPAECs), thus playing a positive role in vascular remodeling related to pulmonary hypertension (PH) [143]. In drug intervention experiments, the traditional Chinese medicine Shenxianshengmai (SXSM) significantly up-regulates SHMT2 expression by activating the CaMKII-AC-PKA signaling cascade, thereby redirecting glutamine metabolism into the TCA cycle. Furthermore, SXSM promotes the TCA cycle and oxidative phosphorylation while significantly inhibiting the apoptotic pathway of cardiomyocytes, thereby improving cardiomyocyte beating function [144].

In summary, SHMT1 and SHMT2 play a multifaceted role in cardiovascular disease and pathology, from embryonic heart development to adult cardiovascular disease, by regulating folate metabolism, oxidative stress, and energy metabolism, among other pathways.

#### SHMT and cerebrovascular diseases

Recent studies on 1C metabolism in stroke have centered on folate metabolism. Evidence indicates that folate deficiency intensifies neuronal autophagy-related death and brain injury following cerebral ischemia-reperfusion, underscoring the significance of folate in neuronal health and its potential implications for stroke prevention [145]. Folic acid mitigates thromboxane A2 release and coagulation factor expression by lowering homocysteine levels. Consequently, this results in a decrease in platelet activation and thrombosis, ultimately leading to the prevention of stroke [146]. A study evaluated the effect of folic acid supplementation on homocysteine reduction and stroke susceptibility. The results of the investigation suggest that folic acid supplementation is advantageous in mitigating the risk of stroke in regions where folic acid fortification is not a viable option [147]. SHMT1 hypermethylation is significantly associated with elevated homocysteine concentrations in patients with ischemic stroke, with elevated SHMT1 methylation levels observed in this population [148]. However, the specific function of SHMT in stroke remains to be elucidated.

### SHMT in inflammation and infection

SHMT-mediated 1C metabolism critically modulates inflammation. The activity of SHMT2 and the availability of serine/glycine have been shown to serve as metabolic hubs, regulating the proliferation of keratinocytes and the inflammatory response in cases of psoriasis. Topical SHMT inhibitors suppress these processes and downregulate disease-associated gene expression [149]. SHMT2 also regulates inflammatory cytokines through its association with the BRISC deubiquitinating enzyme [61]. In osteoarthritis (OA), SHMT2 expression is elevated in chondrocytes, and miR-370 affects the pathological process of osteoarthritis by altering 1C metabolism and DNA methylation through negative regulation of SHMT2 expression [150]. Notably, SHMT2 preserves CD8⁺ T-cell function during HIV-1 infection by mitigating ROS-induced senescence [151]. These findings underscore SHMT’s pleiotropic roles in inflammatory and infectious diseases.

**Fig. 4:**
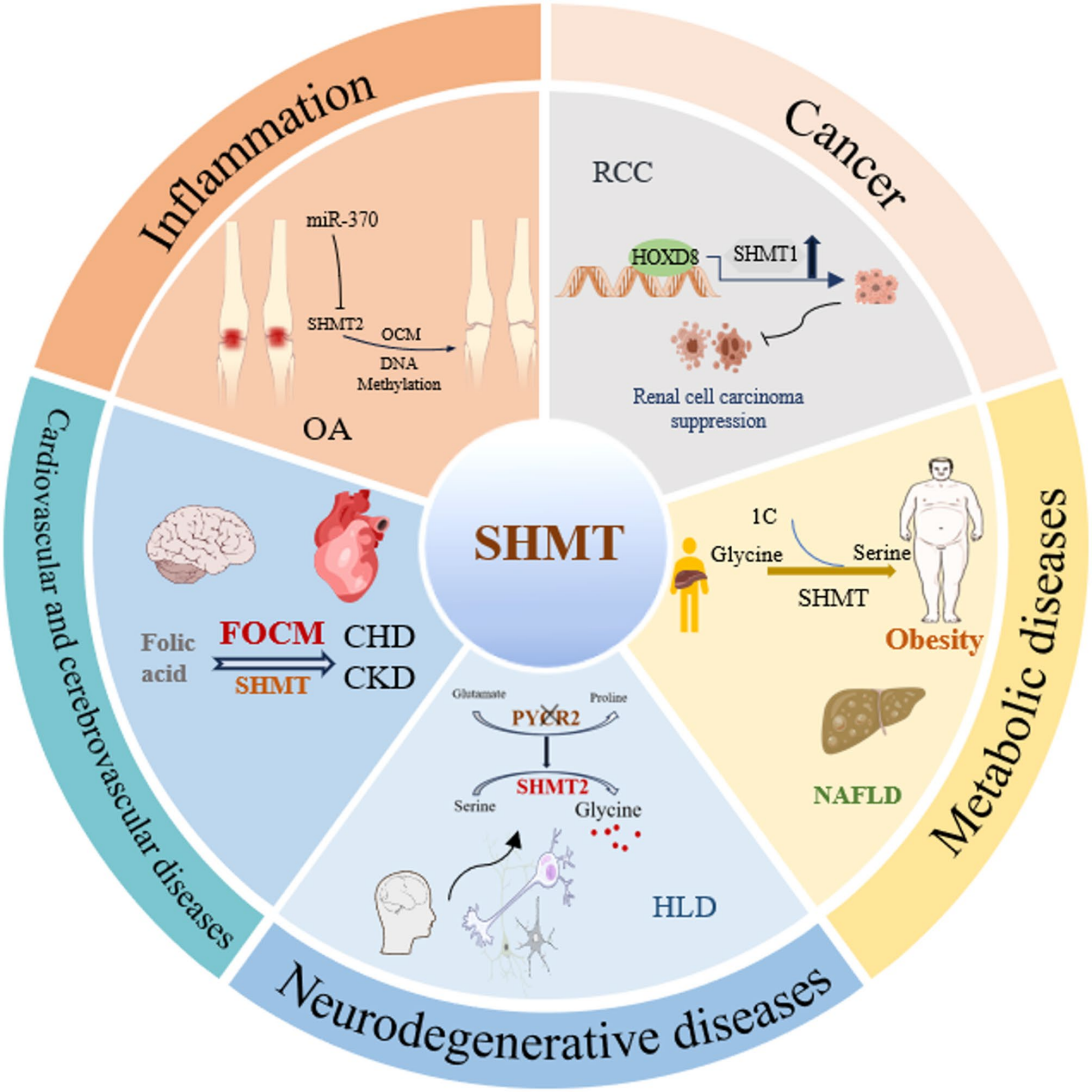
Role of SHMT in disease. SHMT1 and SHMT2 are implicated in cancer, metabolic diseases, neurodegenerative diseases, cardiovascular diseases, and inflammation through their involvement in one-carbon metabolism and amino acid metabolism

Overall, SHMT plays important roles in various pathological conditions and participates in the occurrence and development of multiple cancers. At the same time, it also exerts multiple regulatory functions in metabolic-associated liver diseases, neurological disorders, cardiovascular and cerebrovascular diseases, and inflammatory diseases. (as shown in Fig. 4).

## Therapeutic potential of SHMT inhibitors in diseases

### Types of SHMT inhibitors

In light of the vital function of SHMT in diverse forms of cancer and the therapeutic potential of its knockdown, there is an urgent need to develop highly selective SHMT inhibitors. The first irreversible inhibitor of SHMT, NSC127755 (with an IC50 of approximately 50 nM), limited its clinical application due to severe adverse effects [152]. 5-meTHF inhibits SHMT1/SHMT2, yet its therapeutic value diminishes due to its tendency to transform into alternative folate analogs in humans [153]. Subsequent research, through the optimization and screening of plant SHMT inhibitors in herbicides, led to the development of highly efficient inhibitors featuring a pyrazolopyran core structure that target plasmodium SHMT [154]. Compound 2.12(1) (patent WO2013182472A1) was optimized on the basis of this structure, resulting in a dual SHMT1/2 inhibitor that demonstrated significant anticancer activity [155]. In 2017, Ducker’s team further optimized the compound to obtain SHIN1, which increased the potency of the inhibition of human SHMT1/2 by more than 100-fold (IC50∼10 nM) [156]. Recent studies have identified the potential use of SHIN1 for the purpose of treating mitochondrial myopathy, encephalopathy, lactic acidosis, and stroke-like episodes (MELAS) [157]. SHIN1 can also induce cell cycle arrest in acute lymphoblastic leukemia (ALL) cells [158].

Beyond pyrazolopyran compounds, antifolate-structured drugs also target SHMT. The present study evaluated the interaction mode between SHMT and pemetrexed (PTX), an antifolate drug that functions by inhibiting several enzymes in the folate-dependent biosynthetic pathway. The process by which SHMT inhibition occurs as a result of PTX exposure was successfully identified in vitro, a finding that emerged through the employment of human recombinant proteins [159]. In 2015, Alessandro Paiardini and colleagues further investigated the interaction mechanisms of SHMT with four antifolate drugs (lometrexol (LTX), nolatrexed, raltitrexed, and MTX), discovering that LTX competitively inhibited SHMT at a low micromolar inhibition constant (Ki) [160]. Subsequent work further established that the antifolate compounds LTX and PTX inhibit SHMT2, with LTX identified as the most potent SHMT2 inhibitor reported to date, followed by PTX [161]. In order to efficiently screen inhibitors, a direct system was established for virtual screening, protein expression, and inhibitor identification for SHMT2, which was used to virtually screen 210 thousand compounds from the Specs database for further screening by in vitro non-competitive kinetic assays catalyzed by SHMT2 monoenzyme [162]. Using this system, compound W478 was identified as a previously unobserved SHMT2 inhibitor, which is distinguished from previously reported inhibitors of SHMT2, such as antifolate analogues and pyrazolopyran derivatives, by virtue of its unique chemical structure. Compound W478 binds to SHMT2 at its folate binding site, suggesting its potential as a highly effective lead compound for the synthesis of SHMT2 inhibitors, which may lead to new treatment options for esophageal cancer [163].

Furthermore, 5-substituted pyrrolo[3,2-*d*]pyrimidine compounds target SHMT2 in mitochondria, SHMT1 in the cytoplasm, and enzymes involved in *de novo* purine biosynthesis. The lead compounds (AGF291, AGF320, AGF347) have demonstrated in vitro anti-tumor efficacy in lung, colon, and pancreatic cancer cells [164]. The anti-tumor effects of AGF347, acting downstream of SHMT2 and purine biosynthesis, include inhibition of mTOR signaling, as well as depletion of GSH accompanied by elevated ROS levels [165]. Further structural optimization led to the discovery of novel first-in-class conformationally flexible pyrrolo[3,2-*d*]pyrimidine antifolate inhibitors, which achieve tumor selectivity through folate receptors (FRs). Compared to the parent compound 1, these inhibitors exhibit a 28-fold increase in SHMT2 inhibition, a 21-fold increase in SHMT1 inhibition, and an 11-fold increase in glycinamide ribonucleotide formyltransferase (GARFTase) inhibition [166]. After entering cells, AGF347 is catalyzed by folylpolyglutamate synthase (FPGS) to conjugate five glutamate residues, forming AGF347-Glu5. AGF347-Glu5 inhibits SHMT2 more than 19-fold more potently than AGF347 [167]. It has been reported that SHIN1 promotes cell growth rather than inhibiting proliferation under hypoxic conditions. Inhibition of SHMT2 by SHIN1 leads to NADH depletion under hypoxia, which in turn results in the accumulation of the TCA cycle intermediate oxaloacetate and aspartate. This transamination further promotes purine biosynthesis and cell growth [57]. Pyrrolo[3,2-*d*]pyrimidine compounds, in addition to inhibiting SHMT2, also inhibit *de novo* purine biosynthesis at multiple steps. Therefore, they may overcome the negative consequences exhibited by SHIN1 under hypoxic conditions [164, 166]. In summary, SHIN1, SHIN2, and AGF347 are well-established inhibitors that can simultaneously target both SHMT1 and SHMT2.

Meanwhile, other drugs such as the antidepressant sertraline can be used as SHMT inhibitors to suppress the growth of breast tumors dependent on serine/glycine synthesis [168]. Metformin acts as a non-catalytic PLP-competitive inhibitor of mitochondrial SHMT2, exerting anticancer effects [169]. 4-Chloro-L-threonine inactivates SHMT in a time- and concentration-dependent manner, acting as both a substrate and a potent inhibitor of SHMT [170]. Glycyrrhetinic acid, by competitively inhibiting SHMT2, downregulates mitochondrial oxidative phosphorylation and fatty acid β-oxidation to limit mitochondrial energy supply, thereby suppressing cancer cell proliferation and tumor growth [171]. Mangiferin acts as a competitive inhibitor of THF, binding favorably to SHMT in a manner similar to THF and inhibiting SHMT activity [172].

### Therapeutic advantages and limitations of SHMT inhibitors

Targeting folate metabolism has been clinically used in cancer therapy for over 70 years [173]. Classical antifolate drugs contain a complete pteridine ring, PABA, and glutamate structures, and are structural analogs of folic acid. They competitively inhibit key enzymes in the folate cycle (such as DHFR, TS, and GARFTase, etc.), thereby blocking nucleotide and amino acid synthesis. Furthermore, FPGS adds glutamate chains to folate and antifolate drugs, causing them to be retained within cells and enhancing their activity, ultimately inhibiting the rapid proliferation of tumor cells [174]. In contrast, SHMT inhibitors (such as the SHIN1 series) adopt novel non-folate scaffolds, enhancing selectivity for SHMT [156]. Targeting the tumor-associated mitochondrial isoform SHMT2 is expected to reduce toxicity to normal cells and overcome drug resistance caused by variations in transporters or FPGS [175].

All clinically used antifolate drugs (such as MTX and PTX) are primarily transported via the reduced folate carrier (RFC) [176]. RFC is widely expressed in normal tissues [177], and the inhibition of normal tissue proliferation by antifolates is the main cause of dose-limiting toxicity and drug resistance. Thus, exploring more tumor-targeted therapeutic approaches remains a major challenge. Novel first-in-class conformationally flexible pyrrolo[3,2-*d*]pyrimidine antifolate inhibitors achieve tumor selectivity through FRs while inhibiting SHMT1/2 [166]. They achieve tumor-selective transport via FRα/β and PCFT, reducing RFC-mediated uptake by normal tissues—this represents a fundamental mechanistic improvement over traditional antifolates (e.g., MTX and PTX). Notably, the antitumor efficacy of AGF347 in xenograft models is superior to that of the conventional chemotherapy drug gemcitabine [164]. The classical mechanism of antifolate drugs involves disruption of 1C metabolism, whereas SHMT inhibitors primarily function by limiting glycine synthesis. In diffuse large B-cell lymphoma (DLBCL), certain cell lines are defective in glycine uptake, resulting in a unique dependence on SHMT activity to meet their glycine requirements. Consequently, targeted inhibition by SHIN1 enhances therapeutic efficacy [156]. Interestingly, some traditional antifolate drugs themselves possess inhibitory activity against SHMT. Lometrexol is currently one of the most potent known inhibitors of SHMT1/2 [160], while the polyglutamated form of PTX can also effectively inhibit SHMT in vivo [159]. This suggests that inhibition of SHMT may be one of the mechanisms through which these classical drugs exert their antitumor effects, and further validates the importance of SHMT as a drug target. The development of novel SHMT inhibitors is precisely a deepening and expansion based on this foundation, with the goal of developing next-generation drugs that are more specific and more effective.

While current research has shown some efficacy of SHMT inhibitors in cancer treatment, the fact that SHMT inhibitors target a central metabolic enzyme present in both normal and tumor cells means that potential toxicity remains one of the greatest challenges on the path to clinical translation. The reaction catalyzed by SHMT, which converts serine and tetrahydrofolate to glycine and 5,10-meTHF, is not only crucial for tumor cells but also essential for all rapidly proliferating normal cells [10]. Intestinal epithelial cells [178], hair follicle cells, and germ cells [179] all depend on 1C metabolism to support their high-frequency DNA replication. There are also potential risks to the central and peripheral nervous systems, as 1C metabolism plays an important role in nervous system development and function maintenance [37, 180]. Glycine itself is also a neurotransmitter; therefore, the potential neurotoxicity of SHMT inhibitors is an issue that requires close monitoring in preclinical and early clinical studies. Furthermore, SHMT inhibitors still require further research to define the optimal therapeutic window and assess long-term safety. To date, SHMT inhibitors have only been validated in vitro and in animal experiments, and clinical trials are needed to advance their clinical application. The complexity of the human tumor microenvironment and metabolism represents one of the key challenges in the clinical translation of SHMT inhibitors.

### Combination therapy of SHMT inhibitors

Given that currently available SHMT inhibitors have not yet entered clinical use, and that cancer cells may compensate for SHMT inhibition through alternative metabolic pathways; coupled with the urgent need to explore new therapeutic strategies to overcome chemotherapy drug resistance, combination therapy strategies represent a major focus of research. Sertraline, as an SHMT inhibitor, when combined with mitochondrial inhibitors (such as the antimalarial artemether), induces G1-S phase cell cycle arrest and produces serine-selective antitumor activity in a mouse xenograft model of breast cancer [168]. Inhibition of GSK3 promotes serine/1 C metabolism and exhibits a significant synergistic effect with SHIN1 in suppressing the proliferation of lung cancer cells [1].

The combined use of SHMT inhibitors with anticancer drugs also exerts synergistic therapeutic effects. Studies have found that circ_0063526 upregulates SHMT2 expression through miR-449a, thereby enhancing the resistance of gastric cancer cells to cisplatin [181]. Additionally, estrogen-related receptor α (ERRα) activates SHMT2 transcription by targeting its promoter region, thereby enhancing breast cancer resistance to lapatinib [182]. These findings indicate that SHMT proteins may be associated with the development of drug resistance in tumor cells. SHIN2, when used in combination with paclitaxel (a first-line non-small cell lung cancer treatment), is also effective in reducing the growth of solid tumors in lung cancer [12]. In HCC, SHMT2 knockdown increases the sensitivity of hepatocellular carcinoma cells to doxorubicin [183]. These findings indicate that SHMT proteins may be associated with the development of drug resistance in tumor cells and suggest the potential of SHMT inhibitors in combination chemotherapy. SHIN2, when used in combination with paclitaxel (a first-line non-small cell lung cancer treatment), is also effective in reducing the growth of solid tumors in lung cancer [12]. SHIN2 and MTX synergize in Notch1-driven primary mouse T-cell acute lymphoblastic leukemia and in vivo patient-derived xenografts, effectively overcoming MTX resistance and improving survival rates [184]. Meanwhile, SHIN1 also reduces leukemia burden in vivo and maintains the efficacy of MTX treatment in vitro [158]. Additionally, in Burkitt lymphoma (BL) models, MTX and other drugs exhibit synergistic effects with SHMT inhibitors [99]. SHMT2 is a key regulator of 5-FU chemotherapy resistance in CRC [104]. Alterations in serine metabolism affect 5-FU sensitivity in both in vitro and in vivo CRC models; notably, 5-FU-resistant CRC cells exhibit a strong serine addiction. Inhibiting SHMT2 activity, thereby interfering with serine availability, enhances the antitumor efficacy of 5-FU in CRC models [103]. Inhibiting the expression of SHMT1 significantly reduced GCSC sphere formation and enhanced the sensitivity of Drosha knockdown gastric cancer cells to 5-FU [73]. Furthermore, SHIN1 synergizes with 5-FU, and the combination treatment induces cell cycle arrest, DNA damage, and cellular senescence by regulating the P53 signaling pathway; achieving cell cycle arrest, thereby alleviating chemotherapy resistance and inhibiting gastric cancer [185]. As the most abundant antioxidant, GSH promotes cancer cell survival and chemoresistance by scavenging excess ROS induced by platinum-based drugs [186]. The combination of AGF347 and cisplatin in cisplatin-resistant EOC leads to the depletion of GSH, glycine, and mitochondrial NADH, thereby producing synergistic inhibition and enhanced antitumor efficacy [84]. This demonstrates that combination therapy with SHMT inhibitors can sensitize chemotherapy, overcome drug resistance, and block metabolic adaptation.

These effects have been observed not only in isolation but also in combination with various anticancer drugs, yielding enhanced outcomes (as shown in Table 1).

**Table 1:** Summary of SHMT inhibitors and their pharmacological properties

SHMT Inhibitor	Target	Key Properties / Mechanisms	Reference
NSC127755	SHMT1/2	First irreversible inhibitor; severe adverse effects limit use.	[152]
5-CH_2_-THF	SHMT1/2	Natural inhibitor; converts to other folates in vivo, limiting clinical applicability	[153]
Compound 2.12(1)	SHMT1/2	Pyrazolopyran-based dual inhibitor with potent anticancer activity	[155]
SHIN1	SHMT1/2	On-target dual inhibition at nanomolar concentrations (IC50 ≈ 10 nM)	[156]
SHIN2	SHMT1/2	Reduces growth of solid tumors (e.g., lung cancer) and T-cell acute lymphoblastic leukemia	[184]
LTX	SHMT2	Antifolate; Most potent competitive SHMT2 inhibitor known.	[161]
PTX	SHMT2	Antifolate; Potent SHMT2 inhibitor.	[161]
Sertraline	SHMT1/2	Antidepressant repurposed as SHMT inhibitor; inhibits serine/glycine-dependent tumor growth.	[168]
Compound W478	SHMT2	Novel scaffold distinct from antifolates/pyrazolopyrans; binds folate site	[163]
AGF347	SHMT1/2	Multi-target antifolate agent inhibiting SHMT1/2 and purine biosynthesis enzymes.	[164]
Novel pyrrolo[3,2-*d*]pyrimidine antifolates	SHMT1/2	Conformationally flexible inhibitors; Compared to AGF347, show 28-fold increased SHMT2 inhibition, 21-fold increased SHMT1 inhibition, and 11-fold increased GARFTase (glycineamide ribonucleotide formyltransferase) inhibition.	[166]
Metformin	SHMT2	Non-catalytic PLP competitive inhibitor of SHMT2	[169]
4-Chloro-L-threonine	SHMT1/2	Inactivates SHMT in a time- and concentration-dependent manner.	[166]
Glycyrrhetinic acid	SHMT2	Competitively inhibits SHMT2	[171]
Mangiferin	SHMT1/2	THF- competitive inhibitor; binds SHMT in a manner similar to THF.	[172]

## Conclusion

As a form of metabolic reprogramming, FOCM has emerged as a prominent research focus in recent years, playing critical roles in the pathogenesis and treatment of diverse diseases. SHMT-mediated 1C metabolism supports physiological processes by supplying precursors for amino acid interconversion and nucleotide synthesis. Additionally, via the folate and methionine cycles, SHMT generates 5,10-meTHF, 10-formyl-THF, SAM, and polyamines—molecules essential for nucleotide biosynthesis and epigenetic regulation. SHMT also maintains serine-glycine homeostasis, which is indispensable for purine, thymidine, methionine, and GSH synthesis, while preserving redox balance through GSH and folate-dependent NADPH production. Thus, a deeper understanding of these pathways could facilitate the precise targeting of cancer-specific vulnerabilities in malignancies such as ovarian, lung, liver, gastric, colorectal, and renal cancers. Research has further explored the regulatory roles of transcription factors, RNA, and post-translational modifications on SHMT activity, which influence cancer initiation, progression, and metastasis. Conventional chemotherapies such as MTX (antifolate) and 5-FU (thymidylate synthase inhibitor) target DHFR and TYMS in the FOCM pathway. However, their broad inhibition of folate metabolism often causes severe adverse effects, owing to the pathway’s critical role in normal cells. Moreover, cancer cells frequently develop folate resistance. Future therapies may overcome these limitations by selectively inhibiting individual FOCM enzymes (e.g., SHMT1/2). Isoform-specific SHMT inhibitors could minimize off-target effects while exploiting cancer cell dependencies. Beyond oncology, SHMT-mediated 1C metabolism regulates hepatic disorders (e.g., NAFLD, steatohepatitis), epidermal inflammation, osteoarthritis, and neurodegenerative diseases (e.g., schizophrenia, HLD, stroke). Advances in SHMT inhibitors and their potential applications in targeted therapies offer novel strategies for disease intervention.

Current research on SHMT-mediated 1C metabolism faces several persistent challenges that warrant further investigation. Firstly, the field requires more advanced metabolite detection systems. Key folate metabolites, including 5,10-meTHF, 10-formyl-THF, and formate are often excluded from routine metabolomic analyses. Failure to quantify these metabolites leads to underestimation of their functional roles in disease pathogenesis. Secondly, the complete scope of reactions and products in 1C metabolism, as well as the specific mechanisms by which certain non-canonical “byproducts” influence disease pathogenesis, remain incompletely understood. Examples include the critical roles of GSH and NADPH in supporting antioxidant defense, and the involvement of SAM in DNA methylation. Thirdly, current drugs targeting 1C metabolism are limited by side effects and resistance to antifolates. Inhibitors targeting SHMT1/2 may overcome resistance to traditional antifolate therapies; however, they are currently only being studied in combination with anticancer agents. Research on their efficacy in other metabolic diseases, as well as their own side effects and resistance mechanisms, remains limited. The translation of these inhibitors into clinical practice remains to be achieved, with significant challenges remaining to be addressed. Future research on SHMT-mediated 1C metabolism will focus on leveraging advanced computer-aided drug design (CADD) to develop novel inhibitors, elucidating their mechanisms of action, and exploring enhanced therapeutic targeting strategies and novel therapeutic approaches.

## Data Availability

Data sharing is not applicable to this article as no datasets were generated or analysed during the current study.

## Abbreviations

SHMT1/2: Serine Hydroxymethyltransferase 1 and 2
1C: One-carbon
3-PG: 3-phosphoglycerate
PHGDH: Phosphoglycerate dehydrogenase
PSAT1: Phosphoserine aminotransferase 1
PSPH: Phosphoserine phosphatase
THF: Tetrahydrofolate
5,10-meTHF: 5,10-methylenetetrahydrofolate
TME: Tumor microenvironment
FOCM: Folate-mediated One-carbon Metabolism
SAM: S-adenosylmethionine
DHFR: Dihydrofolate reductase
dTMP: Deoxythymidine monophosphate
UMFA: Unmetabolized folic acid
MTR/MS: Methionine synthase
TYMS: Thymidylate synthase
dUMP: Deoxyuridine monophosphate
MTHFR: methylenetetrahydrofolate reductase
5-meTHF: 5-methyltetrahydrofolate
Met: Methionine
MAT: Methionine adenosyltransferase
MT: Methyltransferase
SAH: S-adenosylhomocysteine
AHCY: Adenosylhomocysteinase
Hcy: Homocysteine
BHMT: Betaine-homocysteine S-methyltransferase
MTHFD1/2: Methylenetetrahydrofolate dehydrogenase 1/2
ALDH1L1/2: 10-formyltetrahydrofolate dehydrogenase 1/2
SSP: Serine synthesis pathway
3-PHP: 3-phosphohydroxypyruvate
3-PS: 3-phosphoserine
Glu: Glutamate
PKM2: Pyruvate kinase M2 isoform
MTFMT: Mitochondrial methionyl-tRNA formyltransferase
fMet-tRNA: Formylmethionyl-tRNA
GART: Phosphoribosylglycinamide formyltransferase
ATIC: 5-aminoimidazole-4-carboxamide ribonucleotide formyltransferase/ IMP cyclohydrolase
NEAAs: Non-essential amino acids
GCS: Glycine cleavage system
SGOC: Serine-glycine-one-carbon
GSH: Glutathione
ADAM10: ADAM metallopeptidase domain 10
APP: Amyloid precursor protein
Aβ: β-amyloid
BRISC: BRCC36 isopeptidase complex
IFNAR1: Interferon alpha receptor 1
DUB: Deubiquitinating enzyme
HIF-1α: Hypoxia-inducible factor-1α
CRC: Colorectal cancer
GSK3: Glycogen synthase kinase 3
NRF2: Nuclear factor erythroid 2-related factor 2
ATF4: Activating transcription factor 4
IGF2BP1: Insulin-like growth factor 2 mRNA-binding protein 1
5'-UTR: 5’-untranslated region
GCSCs: Gastric cancer stem cells
PTMs: Post-translational modifications
HDAC11: Histone deacetylase 11
ROS: Reactive oxygen species
EOC: Epithelial ovarian cancer
NSCLC: Non-small cell lung cancer
HDM2: Human double minute 2
5-FU: 5-fluorouracil
SETTLE: Spindle Epithelial Tumor with Thymus-Like Differentiation
LGG: Low-grade glioma
IDH3α: Isocitrate dehydrogenase 3α
CRLM: Colorectal cancer liver metastasis
EMT: Epithelial-mesenchymal transition
RCC: Renal cell carcinoma
HCC: Hepatocellular carcinoma
NOX1: NADPH oxidase 1
SDH: Succinate dehydrogenase
MASH: Metabolic dysfunction-associated steatohepatitis
NAFLD: Non-alcoholic fatty liver disease
NASH: Non-alcoholic steatohepatitis
MASLD: Metabolic dysfunction-associated steatotic liver disease
BCAT1: Branched chain amino-acid transaminase 1
MS: Multiple sclerosis
PYCR2: Pyrroline-5-carboxylate reductase 2
PPI: Pre-pulse inhibition
CHD: Congenital heart disease
CVD: Cardiovascular disease
HRV: Heart rate variability
CKD: Chronic kidney disease
VSMCs: Vascular smooth muscle cells
DKK1: Dickkopf 1
PH: Pulmonary hypertension
SXSM: Shenxianshengmai
MELAS: Mitochondrial myopathy, encephalopathy, lactic acidosis, and stroke-like episodes
ALL: Acute lymphoblastic leukemia
LTX: Lometrexol
PTX: Pemetrexed
MTX: Methotrexate
GARFTase: Glycinamide ribonucleotide formyltransferase
FPGS: Folylpolyglutamate synthase
RFC: Reduced folate carrier
DLBCL: Diffuse large B-cell lymphoma
ERRα: Estrogen-related receptorα
BL: Burkitt lymphoma
HLD: Hypomyelinating leukodystrophy
CADD: Computer-aided drug design
